# The Effect of Combining Blood Flow Restriction with the Nordic Hamstring Exercise on Hamstring Strength: Randomized Controlled Trial

**DOI:** 10.3390/jcm13072035

**Published:** 2024-04-01

**Authors:** Necdet Eray Pişkin, Gönül Yavuz, Zait Burak Aktuğ, Monira I. Aldhahi, Sameer Badri Al-Mhanna, Mehmet Gülü

**Affiliations:** 1 Department of Movement and Training Sciences, Faculty of Sports Sciences, Nigde Omer Halisdemir University, Niğde 51240, Türkiye; eraypiskin@ohu.edu.tr; 2 Department of Physical Education and Sports, Faculty of Sports Sciences, Kahramanmaras Sutcu Imam University, Kahramanmaras 46050, Türkiye; gonulyavuz@ksu.edu.tr; 3 Department of Physical Education and Sports, Faculty of Sports Sciences, Nigde Omer Halisdemir University, Niğde 51240, Türkiye; zaktug@ohu.edu.tr; 4 Department of Rehabilitation Sciences, College of Health and Rehabilitation Sciences, Princess Nourah bint Abdulrahman University, P.O. Box 84428, Riyadh 11671, Saudi Arabia; 5 Department of Physiology, School of Medical Sciences, University Sains Malaysia, Kubang Kerian 16150, Kelantan, Malaysia; sameerbadri9@gmail.com; 6 Department of Sports Management, Faculty of Sport Sciences, Kirikkale University, Kirikkale 71450, Türkiye

**Keywords:** blood flow restriction, nordic hamstring exercise, peak torque, bilateral deficit, training volume

## Abstract

**(1) Background:** It is a matter of curiosity what effect the blood flow restriction (BFR) method, which is usually combined with low-intensity resistance exercises, will have when used with high-intensity eccentric exercises. **(2) Methods:** The present study examined the effects of combining BFR with nordic hamstring exercises (NHEs) on hamstring muscle strength, bilateral deficit (BLD), and training volume. Thirty young female volleyball players, who trained three times a week, participated voluntarily in the study. These players were stratified into three groups, each comprising ten individuals: a control group (CG), an NHE group, and an NHE + BFR group. Hamstring muscle strength and BLD values were determined using an H-BORD device, while training volume was measured in terms of sets and repetitions. **(3) Results:** Statistical analysis revealed that there were no statistically significant differences in non-dominant and dominant leg peak torque parameters in the exercise groups (F = 2.65; *p* = 0.097; η_p_^2^ = 0.17; F = 1.15; *p* = 0.0334; η_p_^2^ = 0.084), while the total training volume was lower in the NHE + BFR group. **(4) Conclusions:** As a result, it was seen that adding the BFR method to NHE did not provide additional gains. However, due to the low training volume of BFR + NHE, it may be recommended to apply BFR together with NHE to athlete groups.

## 1. Introduction

Resistance exercises are commonly used as the primary method for stimulating muscle adaptations and enhancing strength. Although the intensity variable in resistance exercises remains a subject of debate, the literature suggests a range of 60−80% of the one-repetition maximum (1RM) intensity for hypertrophy development [1]. Trainers and physical therapists dedicated to achieving physiological adaptations and optimizing performance consider numerous variables and stay abreast of novel methods when formulating training programs [2]. In recent years, the blood flow restriction (BFR) method has gained popularity, especially in the context of strength development [3], and has become a method frequently integrated into training regimens [4,5]. The underlying principle of this method is to achieve maximum benefits through use of low-intensity exercises. The BFR method is applied by using cuffs that are wrapped around the thigh region for the lower extremity and around the proximal part of the arm for the upper extremity. With the pressure exerted by these cuffs, arterial blood flow is restricted without completely cutting it off, thereby limiting venous return and creating an anaerobic environment similar to that achieved during high-intensity exercises [6]. The preferred intensity for loading, typically falling within the range of 20−40% of the 1RM, is commonly associated with resistance exercises. The existing literature has demonstrated that this approach can yield results comparable to those achieved with high-intensity resistance exercises [7]. However, it is important to consider that the BFR method may not provide sufficient stimulation, especially in players in good physical condition. Therefore, it is recommended that the BFR method not serve as the primary strength program but rather be integrated with traditional resistance exercises [5,8].

Cook et al. (2014) contributed a unique perspective to the literature by exploring the application of the BFR method during high-intensity exercises in well-trained players. The study indicated that a combination of the BFR method with high-intensity exercises could lead to greater gains in the athlete population compared to low-intensity BFR applications [9]. One specific exercise, the nordic hamstring exercise (NHE), is characterized by its eccentric and high-intensity nature [10]. Notably, the NHE’s slow eccentric contraction places significant stress on the myosin heads bound to actin, resulting in their separation and the subsequent elongation of cross-bridges. This mechanism serves as a stimulus for muscle activation, distinguishing it from concentric exercises [10,11]. While there is a limited number of studies in the literature regarding the combination of the BFR method with high-intensity exercises, there exists a curiosity about the potential effects of applying the NHE at maximal intensity and eccentric contraction when combined with BFR in trained players. In light of all this information, the intended aim of this study is to examine the effect of combining the BFR method with the NHE on hamstring muscle strength in young female volleyball players. We hypothesized that combined NHE and BFR application would induce a superior effect on muscle strength and lower the workload (training volume) compared to the NHE-only group.

## 2. Materials and Methods

### 2.1. Study Design and Participants

In determining the sample size, the ‘Test family: *t* test; Statistical test: Means: Difference between two dependent means (matched pairs)’ was used. It was estimated that the force value of 219 ± 38 would be 243 with a 10% difference. With a 5% error rate and 80% power, it was calculated using G*power 3.1.9.2 program that at least 24 participants are required to participate in the study. At the beginning of the study, a total of 45 young female players playing volleyball were reached. After applying the exclusion criteria (those with a history of lower extremity injury [*n* = 6], those with a current hamstring muscle injury [*n* = 4], and those who declined to participate [2]), a total of 12 players were excluded at the initial stage of the study and the research was started with a player group consisting of 33 young female volleyball players (NHE [*n* = 11], NHE + BFR [*n* = 12], CG [*n* = 10]). At a later stage, 3 more participants (NHE [*n* = 1], NHE + BFR [*n* = 2]) who were determined not to participate in training regularly were excluded from the study. The process was followed with 10 participants in each group and analyses were carried out by performing post-test measurements. These players held licenses for at least three years, underwent training sessions three days a week, engaged in competitions one day a week, and had no previous lower extremity injuries in the past three months (Table 1). The study obtained ethical approval from the Non-Invasive Clinical Research Ethics Committee of Nigde Omer Halisdemir University with decision number 2023/27 dated 11 May 2023. This study was conducted in accordance with the principles of the Helsinki Declaration. Prior to measurements, participants were provided with a detailed presentation about the study procedure and informed consent forms were obtained from their parents.

### 2.2. Study Procedure

**Figure 1:**
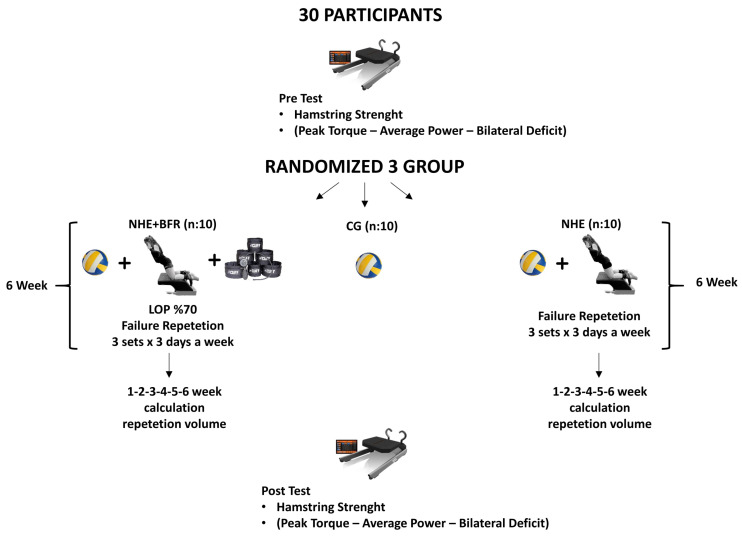
Study design.

A total of thirty women volleyball players volunteered to participate in the study and they were stratified into three groups: NHE group (*n* = 10), NHE + BFR group (*n* = 10), and CG (*n* = 10). The CG continued their regular volleyball training while the other two groups, in addition to volleyball training, performed NHE on three designated days of the week. The NHE + BFR group, unlike the NHE group, performed the exercises in combination with the BFR method. The study was completed in a 9 week period with pre-test and post-test measurements. Measurements were taken in the first week and the last week. The training programs were implemented for 6 weeks, excluding the 1-week anatomical adaptation period. The training volume was calculated by multiplying the number of sets by the number of repetitions in the NHE and NHE + BFR groups during the 6 weeks of training. While calculating the training volume, the steps described in the exercise protocols section were observed by the researcher and noted based on the number of repetitions successfully performed by the participant. In order to determine the specific effects of the NHE in the study, the training loads and rest intervals of all participating players during their routine volleyball training sessions were set to be similar to each other (Figure 1). In addition, routine volleyball training was based especially on technical and tactical exercises during this period.

### 2.3. Evaluation of Hamstring Muscle Strength

The hamstring muscle strength (peak torque), average power, and bilateral deficits (BLD) were assessed using the H BORD device (IVMES, TR, Ankara, Turkey). The reliability and validity tests of the new portable nordic hamstring test device were performed [12]. Additionally, the strength tests were conducted on a day when the players did not engage in any exercise for the past 24 h and completed their meals at least three hours prior to the test. Players underwent both pre-tests and post-tests in the same order, time, and conditions.

### 2.4. Applied Tests

**Figure 2:**
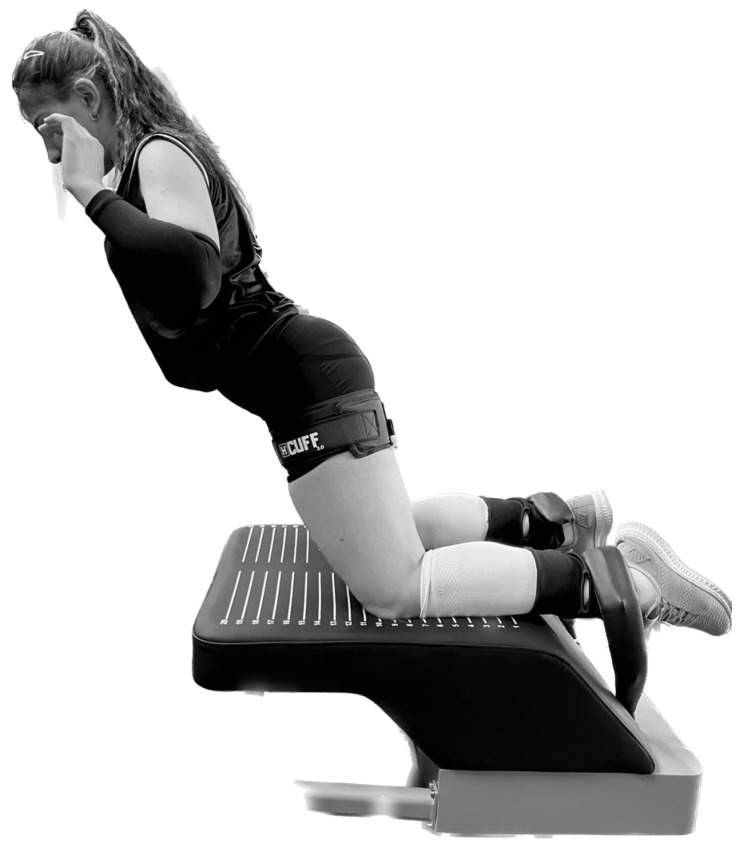
Measurement of hamstring muscle strength.

The hamstring muscle strength of the participants was evaluated with the H BORD device. The ankles of the participants were fixed with bands, and it was confirmed that the body of the H BORD device started moving with both knees, with the body in the same plane as the knee, and the arms in the air with the palms facing forward (Figure 2). Afterwards, the participants continued the movement until the last point they could reach in this position, keeping the knees fixed on the mechanism and slowly letting themselves go forward, without changing their position, with the body in the same plane as the knee. If the participant was able to do so, she came back to the starting point without touching anything and continued the second attempt without interruption. If the participant could not return to the starting position after the forward swing movement, the swing continued until both palms touched the ground, and she took the position again for the second trial by using her hands for support. During this movement, the measurement was calculated in Newton meters (Nm) using the sensors located on the ankles of the H BORD device, and the peak torque of the hamstring, average power, and the BLD values of both legs were recorded. The best grade out of two attempts was included in the study.

### 2.5. Exercise Protocols

#### 2.5.1. NHE Protocol

In the study, players underwent the NHE using the H BORD device. Anatomical adaptation training was carried out the week after the pre-test was applied to the players to ensure the correct execution of the movement. This period was excluded from the experimental process, and subsequently, a 6 week normal training period was started. This training was conducted three days a week. To prevent any misleading increase in training volume due to the incorrect application of the NHE exercise, a 40-degree angle was marked next to the exercise device, and researchers verified this angle as a reference. The number of repetitions performed by the players when they reached this predetermined angle was counted as a successful repetition. The repetitions continued as long as the participant followed the determined steps at a 40-degree angle. The number of repetitions in which the participant could not reach a 40-degree angle was considered unsuccessful, and when this situation was repeated 2 times in a row, the number of successful repetitions until this point was recorded by the researcher. At the specified angle, players were allowed to use their arms and hands to dampen the fall and allow their chest to touch the surface, and immediately returned to the starting position by pushing the ground with their hands [13]. Two groups, the NHE and NHE + BFR groups, performed NHE in the form of three sets of repetitions until failure with a 2 min rest between sets. This exercise protocol was conducted three days a week after the warm-up protocol, specifically on days without volleyball training for six weeks.

#### 2.5.2. NHE + BFR Protocol

An H+ Cuff brand (Hplus Cuff Curve 2.0, Innovative Therapy Solutions, LLC, Santa Clara, CA, USA) curve series cuff was used for the BFR method. It is a 10 cm wide pneumatic cuff type with manual pressure control, cuff pressure ranges of up to 300 mmHg, a precision measuring pump with ±3 mmHg accuracy, and a hand indicator with smart valve technology.

It has been stated that the absolute pressure that occurs after the cuff is attached to the circumference of the limb in exercises performed with the BFR method may lead to a vascular restriction in different degrees from person to person, depending on individual blood pressure, anthropometric characteristics, and cuff width [14]. Considering this situation, LOP was determined with the help of Doppler to determine the participant’s limb occlusion pressure (LOP). The cuff was tied to the proximal part of the participants’ thigh in the position where the motion form would be applied, and it was inflated with an inflation pump to 50 mmHg for 30 s and then reset for 10 s. Subsequently, the inflation–deflation cycle was continued with increments of 10–20 mmHg, gradually increasing the pressure until the pulse was no longer detectable. During this process, the audible signal of the pulse was listened to using a Doppler probe placed on the tibialis posterior artery. The LOP was determined as the point where the audible pulse signal was no longer detected. In the NHE + BFR group, the exercise sessions were performed at a number of repetitions equivalent to 70% of the individually determined LOP, up to three sets until failure with a 2 min rest between sets. The commonly used repetition scheme in the BFR method is typically 30-15-15-15, which has been reported to provide sufficient repetition volume for most individuals to induce the necessary adaptations [6]. However, in our study, we employed a repetition method where the participants performed repetitions until the failure set, reaching a point where they could no longer complete a repetition [15,16,17]. It has been suggested that performing repetitions until a failure set is necessary to induce strength adaptations in certain populations, such as players [6].

### 2.6. Analysis of Data

In this study, the normal distribution assumption of quantitative variables was examined through visual methods (histograms and probability plots) and analytical methods (Shapiro–Wilk test). As the quantitative variables exhibited a normal distribution, they were expressed in terms of mean and standard deviation. To analyse the results of different protocols (NHE, NHE + BFR, and CG), as well as pre and post-test measurements and the protocol by time interaction effect, a repeated-measures two-way ANOVA test was employed. Mauchly’s sphericity test was used to assess the homogeneity of variances, and a Greenhouse–Geisser correction was applied as needed. Partial eta squared (ηp^2^) values were calculated to determine the effect size between groups. ηp^2^ was examined as an index of effect size interpreted as 0.01 = small, 0.06 = medium, and 0.14 = large. In the case of statistically significant differences among the study protocols, multiple comparison analyses were conducted using the Tukey method. A significance level of *p* < 0.05 was considered.

## 3. Results

In the exercise groups, a statistically significant difference was found from pre-test to post-test in the dominant leg peak torque parameter (Time F = 52.31; *p* = 0.000, η_p_^2^ = 0.68), while no difference was observed in the CG. There was no statistically significant difference between the groups (F = 2.65; *p* = 0.097; η_p_^2^ = 0.17). A statistical difference was found in the group by time interaction. According to the Bonferroni correction, this difference is attributed to a significant increase in the NHE and NHE + BFR groups compared to the CG (F = 7.54; *p* = 0.003; η_p_^2^ = 0.38).

A statistically significant difference was observed in the non-dominant leg peak torque parameter when comparing pre-test and post-test measurements across all groups (Time F = 87.27; *p* = 0.000, η_p_^2^ = 0.777). However, there was no statistically significant difference observed among the groups themselves (F = 1.15; *p* = 0.0334; η_p_^2^ = 0.084). Furthermore, a significant statistical difference was identified in the interaction between group and time. Applying the Bonferroni correction revealed that this difference is primarily attributed to a significant increase in the NHE + BFR group compared to the CG (F = 6.03; *p* = 0.007; η_p_^2^ = 0.326). The findings of the pre-test to post-test comparisons in the dominant leg average power parameter in the exercise groups were statistically significant (Time F = 62.56; *p* = 0.000, η_p_^2^ = 0.71), while no difference was observed in the CG. There was no statistically significant difference between the groups (F = 3.097; *p* = 0.063; η_p_^2^ = 0.199). A statistical difference was found in the group by time interaction. According to the Bonferroni correction, this difference is attributed to a significant increase in the NHE and NHE + BFR groups compared to the CG (F = 7.54; *p* = 0.003; η_p_^2^ = 0.38).

A statistically significant difference has been found between the pre-test and post-test measurements in the non-dominant leg average power parameter in the exercise groups (Time F = 77.75; *p* = 0.000, η_p_^2^ = 0.75), with no discernible variation detected in the CG. Notably, no statistically significant differences manifested among the groups themselves (F = 1.65; *p* = 0.211; η_p_^2^ = 0.117). Conversely, a statistical difference was found in the group by time interaction. According to the Bonferroni correction, this difference is attributed to a significant increase in the NHE and NHE + BFR groups compared to the CG (F = 9.88; *p* = 0.001; η_p_^2^ = 0.44).

A statistically significant difference has been found from pre-test to post-test in the bilateral difference parameter in the NHE + BFR group (Time F = 8.554; *p* = 0.007, η_p_^2^ = 0.25), while no difference was observed in the NHE and CG. There was no statistically significant difference between the groups (F = 0.933; *p* = 0.407; η_p_^2^ = 0.07). No statistically significant difference was found in the group by time interaction (F = 0.216; *p* = 0.792; η_p_^2^ = 0.018). When looking at the percentage (%) improvement levels, the highest improvement is observed in the NHE + BFR group (Table 2).

**Figure 3:**
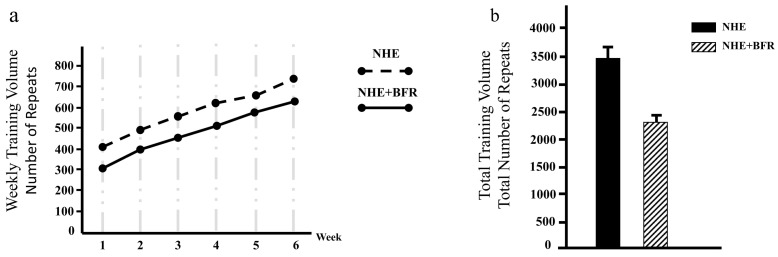
The training volume of NHE and NHE + BFR groups. The training volume data are displayed as the number of repetitions. (**a**) Weekly training volume; (**b**) 6 week total training volume.

When examining the training volume, a linear increase in the number of repetitions was observed from the first week to the last week in both the NHE and NHE + BFR groups (Figure 3a). At the end of the 6 week period, the total training volume was determined as 3484 repetitions in the NHE group and 2317 repetitions in the NHE + BFR group (Figure 3b).

## 4. Discussion

The precise assessment and enhancement of muscle strength balance holds considerable significance in the realm of enhancing athletic performance, devising effective training regimens, mitigating injuries stemming from muscular deficiencies in players, and aiding in post-injury rehabilitation [18]. In this regard, many exercises have been incorporated to target the development of hamstring muscles. Among these exercises, the NHE is known for its effectiveness in increasing hamstring muscle strength and reducing hamstring injury incidence [19,20].

### 4.1. Peak Torque Average Power

In this study, the NHE was implemented in conjunction with the BFR method, and its impact on strength enhancement was meticulously assessed. It revealed a noteworthy disparity in the increases observed in peak torque and average power within both the NHE + BFR and NHE groups, whereas no discernible difference emerged in the CG. According to the Bonferroni correction, this difference is attributed to a significant increase in the NHE and NHE + BFR groups compared to the CG. The addition of the BFR method to the NHE did not result in a higher level of improvement in peak torque and average power increases.

In the context of resistance exercises conducted using BFR methodology, existing literature primarily recommends employing a load intensity falling within the range of 20% to 40% of an individual’s one-repetition maximum (1RM). This specific intensity range has been associated with positive outcomes [6]. Conversely, it has been observed that load intensities below 20% tend to result in minimal hypertrophic effects [21]. However, studies have also been conducted using load intensities above 50% of one RM, and conflicting results exist in the literature regarding this matter. Cook et al. (2014) stated that applying the BFR method at high intensities may yield greater gains compared to low-intensity BFR methods in trained players [9]. Conversely, other research has indicated that load intensities exceeding 50% of the 1RM do not significantly augment the effects of the BFR method [22]. Furthermore, an investigation discovered that performing squat exercises using the BFR method at 80% of the 1RM did not yield additional strength improvements [15]. Our study’s findings align with the aforementioned research, as the BFR exercises implemented in our study were executed at maximal intensities. Both the NHE group and the NHE + BFR group exhibited increased hamstring strength. This suggests that employing the BFR method at maximal effort may suffice for achieving desired strength enhancements, and the incorporation of BFR may not significantly contribute to additional strength gains. The observed discrepancies in the literature may, in part, be attributed to various application variables within the BFR method, including cuff type, cuff pressure, exercise selection, and others.

### 4.2. Bilateral Deficit

In this study, another parameter examined is the BLD. An increase in the BLD can lead to a decrease in sports performance and an increase in the risk of sports injuries. This is often due to the underutilization or inadequate training of certain body parts or sides [23]. In the BLD measurements, a statistically significant improvement in favour of the post-test was only observed in the NHE + BFR group compared to their pre-test. However, no significant difference was found between the groups. While there are limited studies in the literature investigating the effects of the BFR method on BLD, these studies have reported similar findings to our study, indicating a decrease in the BLD ratio [24,25].

It is believed that the lack of a significant improvement in the BLD ratio in the NHE group in our study may be due to the exercise being applied concentrically to both the dominant and non-dominant sides. In a concentric exercise, the dominant side may be more active in order to preserve strength compared to the non-dominant side. On the other hand, the significant improvement observed in the NHE + BFR group may be related to the recruitment of muscle fibre types in the limb experiencing strength loss, which is one of the development mechanisms of the BFR method. Due to the restrictive cuff pressure in the BFR method, the by-products of muscle contractions such as H+ ions, ATP, and inorganic phosphates that cannot exit the limb through the venous system [26] can lead to early neuromuscular fatigue [27,28]. In the presence of this increase in the neuromuscular fatigue, high-threshold motor units are active to maintain muscle strength and complete the set. Therefore, the restriction applied in the BFR method leads to a greater need for the activation of more motor units to compensate for the loss of strength caused by the lack of oxygenation in the affected area [6]. The activation of type II fibres seems to play a significant role in the observed strength development with the BFR method [29,30]. It is thought that the BFR method can produce more improvement in the non-dominant side, which has a greater loss of strength during NHE compared to the dominant side, thanks to the mechanism described above, and this situation will result in an improvement in BLD. In the CG, although there is no statistically significant difference between the pre-test and the post-test measurements, the difference is 48.0%. This reveals that there was a high level of difference in a few people in the CG in the post-test, but this situation was not reflected in the general group.

### 4.3. Training Volume

Another parameter examined in the study is the training volume. It was stated that the effects of training volume should be taken into account when interpreting the data on hypertrophy results in strength training. Training volume has been identified as a significant driver of muscle hypertrophy, following a dose–response relationship [31]. It has been suggested that lower-intensity exercises result in higher training volumes compared to higher-intensity exercises [1]. In our study, both groups were evaluated under equal conditions in terms of volume by implementing repetitions until failure. The training volume in the study was calculated based on the number of sets multiplied by the number of repetitions due to the nature of the NHE exercise being performed with body weight. When examining the training volume data of the NHE + BFR and NHE groups, a linear increase was observed in both groups from the first to the last week. In many studies investigating the NHE, it has been reported that the applied number of repetitions reaches an average of 8 to 12 repetitions towards the end of the training [10]. In our study, this increase was observed at a lower level in the NHE + BFR group compared to the NHE group (Figure 3).

There are two studies in the literature that support this finding. Libardi et al. (2015) compared a high-intensity exercise group with a BFR + low-intensity exercise group in a leg press exercise targeting the lower extremities [32]. In this study, exercise volume (sets × repetitions × lifted weight) increased in both groups over 1–12 weeks. This increase was significantly lower in the BFR group by 41% in the first 6 weeks and 34% in the second 6 week period. Another study that supports our findings compared four groups (low intensity, moderate intensity + BFR, high intensity + BFR, high intensity). It was stated that exercise volume (sets × repetitions × lifted weight) increased in all groups. When examining the weekly increase in exercise volume, it was observed that the high-intensity exercise group without the BFR had the highest level of exercise volume, while the groups using the BFR experienced a decrease in exercise volume due to increased cuff pressure.

When a certain volume is reached, maximum protein synthesis occurs during exercise. This is dependent on the protein synthesis, metabolic stress, or the ability of exercise volume to activate high-threshold motor units. It has been noted that if exercise volume is sufficient, protein synthesis can occur regardless of exercise intensity. In the BFR method, even though exercise intensity is low, the pressure and stress created by the cuff lead to neuromuscular fatigue and an early sense of fatigue [29]. The lower exercise volume observed in the NHE + BFR group in our study can be explained by the mechanism of neuromuscular fatigue [27]. While both groups completed the number of repetitions until failure, it is thought that the BFR method affects fatigue times depending on the mechanism mentioned above. Thus, it is believed that the perceived fatigue determined by the number of repetitions occurred earlier in the NHE + BFR group due to the early onset of neuromuscular fatigue. Additionally, it has been reported that reducing the repetition volume of the NHE does not have a negative effect on strength compared to high-volume training groups. These findings support the idea that lower NHE volumes may be more suitable for players, potentially reducing the risk of hamstring injuries and improving intervention compliance [10].

## 5. Conclusions

This study found that implementing the BFR method during the NHE did not result in additional strength gains in players. However, upon a closer examination of training volume, it became evident that the NHE + BFR group achieved similar strength development with fewer repetitions compared to the NHE group. Consequently, the BFR method was found to produce equivalent strength gains with a reduced workload. This observation holds particular significance, especially during transitional phases of annual training planning for athlete groups or in periods characterized by exceptionally high training intensity. Incorporating exercises with the BFR method, which involves lower training volumes and reduced fatigue, may be a beneficial recommendation. This approach can enhance training variety and facilitate multidimensional development.

The study’s findings are expected to provide valuable insights for athlete populations that have increasingly adopted the BFR method in recent years. It is worth noting that the application of the BFR method to various populations and the presence of numerous variables (such as cuff type, cuff width, pressure determination method, exercise intensity, number of repetitions, frequency, movement structure, type of combined exercises, and materials used) have led to diverse outcomes in the literature, even when addressing the same objectives. While many studies involve the application of maximal loads using external weights, our study’s lack of significant differences between groups in maximal loading with body weight may be attributed to the aforementioned variables. Therefore, it is suggested that future studies with a methodology similar to ours consider varying these factors, as this could lead to different outcomes. Consequently, further research in this area is essential to expand our understanding and knowledge.

## 6. Limitations

The main limitation of the study is that only volleyball branch and female athletes were included in this study.

It is thought that there is a need to conduct similar studies on different age groups and men in different sports branches.

## Figures and Tables

**Table 1:** Demographic variables of the participants.

	NHE	NHE + BFR	CG
Age (years)	15 ± 0.73	15 ± 0.78	15 ± 0.70
Height (cm)	166 ± 6.36	163 ± 4.40	160 ± 5.38
Body weight (kg)	57.30 ± 7.11	52.80 ± 6.92	56.20 ± 9.24

**Table 2:** Dominant and non-dominant hamstring peak torque, average strength development, and bilateral difference in the groups.

Variable	Pre	Post	Δ	%	Two-Way Repeated ANOVA			
	M ± SD	M ± SD	T_B_-T_end_	T_B_-T_end_	Time	Group	Time*Group	Post Hoc Test
Dominant Leg Peak Torque (Watt)								
NHE	144.6 ± 48.9	220.9 ± 30.5 *	76.3 ± 18.0	52.8	F = 52.31	F = 2.56	F = 7.54	NHE + BFR > CG
NHE + BFR	165.8 ± 38.2	241.1 ± 52.6 *	75.3 ± 14.3	45.4	***p* < 0.000**	*p* < 0.097	***p* < 0.003**	NHE > CG
CG	160.1 ± 28.5	171.6 ± 34.0	11.5 ± 5.5	7.1	η_p_^2^ = 0.68	η_p_^2^ = 0.17	η_p_^2^ = 0.38	
Non-dominant Leg Peak Torque (Watt)								
NHE	135.1 ± 51.8	216.1 ± 26.2 *	81.0 ± 25.6	59.6	F = 87.27	F = 1.15	F = 6.03	NHE + BFR > CG
NHE + BFR	146.4 ± 38.6	228.4 ± 46.3 *	82.0 ± 7.7	55.9	***p* < 0.000**	*p* < 0.334	***p* < 0.007**	
CG	148.3 ± 22.7	177.4 ± 35.2 *	29.1 ± 12.5	19.6	η_p_^2^ = 0.777	η_p_^2^ = 0.084	η_p_^2^ = 0.326	
Dominant Leg Average Power (Watt)								
NHE	135.5 ± 42.3	208.6 ± 27.2 *	73.1 ± 15.1	53.9	F = 62.56	F = 3.097	F = 9.75	NHE + BFR > CG
NHE + BFR	147.5 ± 30.7	229.9 ± 51.9 *	82.4 ± 21.2	55.9	***p* < 0.000**	*p* < 0.063	***p* < 0.001**	NHE > CG
CG	145.2 ± 31.8	155.6 ± 34.6	10.4 ± 2.8	7.6	η_p_^2^ = 0.71	η_p_^2^ = 0.199	η_p_^2^ = 0.44	
Non-dominant Leg Average Power (Watt)								
NHE	127.7 ± 46.4	201.2 ± 24.6 *	73.5 ± 21.8	57.5	F = 77.75	F = 1.65	F = 9.88	NHE + BFR > CG
NHE + BFR	137.2 ± 29.0	217.3 ± 49.9 *	80.1 ± 20.9	58.4	***p* < 0.000**	*p* < 0.211	***p* < 0.001**	NHE > CG
CG	142.2 ± 23.7	157.1 ± 43.5	14.9 ± 19.8	10.5	η_p_^2^ = 0.75	η_p_^2^ = 0.117	η_p_^2^ = 0.44	
Bilateral Difference (Difference post–pre)								
NHE	12.3 ± 5.7	9.1 ± 8.9	−3.2 ± 3.2	26.0	F = 8.554	F = 0.933	F = 0.236	
NHE + BFR	13.9 ± 8.1	8.0 ± 6.0 *	−5.9 ± 2.1	42.4	***p* < 0.007**	*p* < 0.407	*p* < 0.792	
CG	10.0 ± 8.1	5.2 ± 3.6	−4.8 ± 4.5	48.0	η_p_^2^ = 0.25	η_p_^2^ = 0.07	η_p_^2^ = 0.018	

Δ = change; Pre = preintervention; Post = postintervention; ηp^2^: partial eta squared. Bold values denote statistical significance at the *p* < 0.05 level. * There is a significant difference between the pre-test and post-test values.

## Data Availability

The data are not publicly available because further research is being conducted and more manuscripts are being prepared. Data for the current study will be available upon reasonable request from the principal investigator or corresponding author.

## References

[1] Schoenfeld B.J., Grgic J., Van Every D.W., Plotkin D.L. (2021). Loading recommendations for muscle strength, hypertrophy, and local endurance: A re-examination of the repetition continuum. Sports.

[2] Haugen T., Seiler S., Sandbakk Ø., Tønnessen E. (2019). The training and development of elite sprint performance: An integration of scientific and best practice literature. Sports Med. Open.

[3] Feng Y., Yin Y., Zhao X., Zhang Y., Zhou Y., Wu Z.A. (2022). bibliometric analysis study of blood flow restriction using CiteSpace. J. Phy. Thera. Sci..

[4] Pignanelli C., Christiansen D., Burr J.F. (2021). Blood flow restriction training and the high performance athlete: Science to application. J. Appl. Physiol..

[5] Wortman R.J., Brown S.M., Savage-Elliott I., Finley Z.J., Mulcahey M.K. (2020). Blood flow restriction training for athletes: A systematic review. Am. J. Sports Med..

[6] Patterson S.D., Hughes L., Warmington S., Burr J., Scott B.R., Owens J., Abe T., Nielsen J.L., Libardi C.A., Laurentino G. (2019). Blood flow restriction exercise: Considerations of methodology, application, and safety. Front. Physiol..

[7] Yamanaka T., Farley R.S., Caputo J.L. (2012). Occlusion training increases muscular strength in division IA football players. Strength Cond. J..

[8] Rolnick N., Schoenfeld B.J. (2020). Blood flow restriction training and the physique athlete: A practical research-based guide to maximizing muscle size. Strength Cond. J..

[9] Cook C.J., Kilduff L.P., Beaven C.M. (2014). Improving strength and power in trained athletes with 3 weeks of occlusion training. Int. J. Sports Physiol. Perform..

[10] Cuthbert M., Ripley N., McMahon J.J., Evans M., Haff G.G., Comfort P. (2020). The effect of nordic hamstring exercise intervention volume on eccentric strength and muscle architecture adaptations: A systematic review and meta-analyses. Sports Med..

[11] Franchi M.V., Reeves N.D., Narici M.V. (2017). Skeletal muscle re-modelling in response to eccentric vs. concentric loading: Morphological, molecular, and metabolic adaptations. Front. Physiol..

[12] Akarcesme C., Cengizel E., Alvurdu S., Bağcı E., Altundağ E., Cengizel Ç.Ö., Şenel Ö. (2024). Reliability and validity of the new portable Nordic hamstring test device (IVMES H-Bord). J. Sports Eng. Tech..

[13] Mjølsnes R., Arnason A., Østhagen T., Raastad T., Bahr R.A. (2004). 10-week randomized trial comparing eccentric vs. concetric hamstring strength training in well-trained soccer players. Scand. J. Med. Sci. Sports.

[14] Lixandrao M.E., Ugrinowitsch C., Berton R., Vechin F.C., Conceição M.S., Libardi C.A., Damas F., Roschel H. (2018). Magnitude of muscle strength and mass adaptations between high-load resistance training versus low-load resistance training associated with blood flow restriction: A systematic review and meta-analysis. Sports Med..

[15] Neto G.R., Santos H.H., Sousa J.B., Júnior A.T., Araújo J.P., Aniceto R.R., Sousa M.S. (2014). Effects of high-intensity blood flow restriction exercise on muscle fatigue. J. Hum. Kinet..

[16] Kacin A., Strazar K. (2011). Frequent low-load ischemic resistance exercise to failure enhances muscle oxygen delivery and endurance capacity. Scand. J. Med. Sci. Sports.

[17] Patterson S.D., Ferguson R.A. (2010). Increase in calf post-occlusive blood flow and strength following short-term resistance exercise training with blood flow restriction in young women. Eur. J. Appl. Physiol..

[18] Miller L.E., Pierson L.M., Nickols-Richardson S.M., Wootten D.F., Selmon S.E., Ramp W.K., Herbert W.G. (2006). Knee extensor and flexor torque development with concentric and eccentric isokinetic training. Res. Q. Exerc. Sport.

[19] Opar D.A., Williams M.D., Shield A.J. (2012). Hamstring strain injuries factors that lead to injury and re-injury. Sports Med..

[20] Bourne M.N., Williams M.D., Opar D.A., Najjar A.A., Kerr G.K., Shield A.J. (2017). Impact of exercise selection on hamstring muscle activation. Br. J. Sports Med..

[21] Buckner S.L., Jessee M.B., Dankel S.J., Mattocks K.T., Mouser J.G., Bell Z.W., Abe T., Bentley J.P., Loenneke J.P. (2020). Blood flow restriction does not augment low force contractions taken to or near task failure. Eur. J. Sport Sci..

[22] Laurentino G., Ugrinowitsch C., Aihara A.Y., Fernandes A.R., Parcell A.C., Ricard M., Tricoli V. (2008). Effects of strength training and vascular occlusion. Int. J. Sports Med..

[23] Aktug Z.B. (2020). Do the exercises performed with a theraband have an effect on knee muscle strength balances?. J. Back Musculoskelet. Rehabil..

[24] Noyes F.R., Barber-Westin S.D., Sipes L. (2021). Blood flow restriction training can improve peak torque strength in chronic atrophic postoperative quadriceps and hamstrings muscles. Arthroscopy, J. Arthrosc. Relat. Surg..

[25] Tennent D.J., Burns T.C., Johnson A.E., Owens J.G., Hylden C.M. (2018). Blood flow restriction training for postoperative lower-extremity weakness: A report of three cases. Curr. Sports Med. Rep..

[26] Loenneke J.P., Fahs C.A., Rossow L.M., Abe T., Bemben M.G. (2012). The anabolic benefits of venous blood flow restriction training may be induced by muscle cell swelling. Med. Hypotheses.

[27] Jessee M.B., Buckner S.L., Mouser J.G., Mattocks K.T., Dankel S.J., Abe T., Bell Z.W., Bentley J.P., Loenneke J.P. (2018). Muscle adaptations to high-load training and very low-load training with and without blood flow restriction. Front. Physiol..

[28] Pearson S.J., Hussain S.R. (2015). A review on the mechanisms of blood-flow restriction resistance training-induced muscle hypertrophy. Sports Med..

[29] Loenneke J.P., Fahs C.A., Wilson J.M., Bemben M.G. (2011). Blood flow restriction: The metabolite/volume threshold theory. Med. Hypotheses.

[30] de Castro F.M.P., Aquino R., Júnior J.A.B., Gonçalves L.G.C., Puggina E.F. (2017). Strength training with vascular occlusion: A review of possible adaptive mechanisms. Hum. Mov..

[31] Schoenfeld B.J., Ogborn D., Krieger J.W. (2017). Dose-response relationship between weekly resistance training volume and increases in muscle mass: A systematic review and meta-analysis. J. Sports Sci..

[32] Libardi C.A., Chacon-Mikahil M.P.T., Cavaglieri C.R., Tricoli V., Roschel H., Vechin F.C., Conceição M.S., Ugrinowitsch C. (2015). Effect of concurrent training with blood flow restriction in the elderly. Int. J. Sports Med..

